# Same-Day Discharge After Left Atrial Appendage Closure and Early Readmissions: A 5-Year National Readmissions Database Analysis

**DOI:** 10.1016/j.shj.2026.101073

**Published:** 2026-06-24

**Authors:** Sangho Hyun, Chia-hung Sze, Aqeel Khanani, Jennifer Trube, Tughral Khan, Avinash Khanna

**Affiliations:** a Department of Internal Medicine, Lakeland Regional Health Medical Center, Lakeland, Florida, USA; b Department of Cardiology, Lakeland Regional Health Medical Center, Lakeland, Florida, USA

**Keywords:** Atrial fibrillation, Left atrial appendage closure, Length of stay, Readmission, Same-day discharge

## Abstract

**Background:**

Same-day discharge (SDD) after left atrial appendage closure (LAAC) may reduce hospitalization burden, but concerns regarding early readmission after discharge remain.

**Methods:**

We performed a retrospective cohort study using the Nationwide Readmissions Database from 2016 through 2020. Adult patients undergoing inpatient LAAC were identified using International Classification of Disease-10 procedure code 02L73DK. The analytic cohort was restricted to length of stay (LOS) 0 or 1 day; SDD was defined as LOS 0 and overnight stay as LOS 1. Survey-weighted modified Poisson regression was used to estimate adjusted risk ratios (RRs) with year-specific hospital-clustered SEs. Models adjusted for demographics, insurance, income quartile, hospital characteristics, hospital LAAC volume, calendar year, and selected comorbidities.

**Results:**

Among the 39,705 index LAAC hospitalizations, 1621 (4.1%) were SDD and 38,084 (95.9%) were overnight stay. SDD increased from 1.6% in 2016 to 7.7% in 2020. Crude 7-day readmission was 2.0% after SDD versus 2.4% after overnight stay, and 30-day readmission was 7.0% versus 7.6%, respectively. After adjustment, SDD was not associated with higher 7-day readmission (RR 0.89; 95% CI 0.62–1.29) or 30-day readmission (RR 1.00; 95% CI 0.82–1.22). Results were similar in missing-data and overlap-weighted sensitivity analyses. Common readmission categories included bleeding/anemia, cardiovascular/thromboembolic causes including heart failure/volume overload, infection/sepsis, and arrhythmia/conduction disorders.

**Conclusions:**

Among selected short-stay inpatient LAAC procedures, SDD increased over time and was not associated with higher early readmission compared with overnight observation. These findings should be interpreted as reflecting outcomes among patients selected for SDD rather than evidence supporting universal SDD after LAAC.

## Highlights

• Same-day discharge after left atrial appendage closure increased from 2016 to 2020.
• Same-day discharge was not associated with higher 7-day readmission.
• 30-day readmission was similar after same-day and overnight discharge.
• Findings support selective same-day discharge after uncomplicated left atrial appendage closure.

## Introduction

Left atrial appendage closure (LAAC) has become an established nonpharmacologic strategy for stroke prevention in selected patients with atrial fibrillation (AF) who are poor candidates for long-term oral anticoagulation. Contemporary US and European AF guidelines, as well as expert consensus statements, support transcatheter LAAC in appropriately selected patients, reflecting the maturation of device-based stroke prevention in clinical practice.^1^, ^2^, ^3^ This role is supported by randomized trial and long-term follow-up data demonstrating LAAC as an alternative to oral anticoagulation in selected patients with nonvalvular AF.^4^, ^5^, ^6^

As LAAC has transitioned from early clinical trials to broad real-world use, procedural safety and care delivery have continued to evolve. Contemporary data from the National Cardiovascular Data Registry Left Atrial Appendage Occlusion Registry demonstrate that real-world LAAC recipients are older and medically complex, yet major in-hospital adverse events are relatively uncommon in modern practice.^7^ These improvements have shifted attention from procedural feasibility alone toward optimizing postprocedural recovery, hospital resource use, and patient-centered discharge pathways.

Same-day discharge (SDD) after LAAC may offer several potential advantages, including improved patient convenience, reduced hospitalization costs, and avoidance of unnecessary overnight observation. Small observational studies have suggested that SDD after LAAC can be feasible in carefully selected patients, particularly when structured postprocedural monitoring and clinical stability assessment are used.^8^^,^^9^ However, patients undergoing LAAC often have advanced age, multiple comorbidities, and elevated bleeding or thromboembolic risk, raising concerns that early discharge could increase readmissions related to bleeding, heart failure, arrhythmia, or infection.

Prior national analyses using the Nationwide Readmissions Database (NRD) have suggested that SDD after LAAC is not associated with higher 30-day readmission and may be associated with lower hospitalization costs.^10^, ^11^, ^12^ However, these studies differed in comparator definitions, with some comparing SDD with all later discharges rather than a strict overnight stay group, and most focused primarily on 30-day readmission rather than very early readmission after discharge. Therefore, using the NRD from 2016 through 2020, we evaluated temporal trends in SDD after LAAC and compared 7-day, 30-day, and cause-specific readmissions among patients discharged the same day versus after overnight observation.

## Methods

### Study Design and Population

We performed a retrospective cohort study using the NRD from 2016 through 2020.^13^ The NRD is an inpatient database designed to support readmission analyses using deidentified patient linkage variables within each calendar year. Because the NRD contains deidentified publicly available data, this study was considered exempt from institutional review board review. This study was conducted in accordance with the Strengthening the Reporting of Observational Studies in Epidemiology (STROBE) guidelines.^14^

We identified adult patients undergoing inpatient percutaneous LAAC using International Classification of Disease (ICD)-10-Procedure Coding System procedure code 02L73DK. For each patient within a calendar year, the first LAAC hospitalization was selected as the index hospitalization. Patients were categorized according to length of stay (LOS) after the index procedure. The primary analytic cohort was restricted to patients with LOS 0 or 1 day to compare SDD with overnight observation. Patients with LOS ≥2 days were excluded because longer hospitalization may reflect complications, delayed recovery, or nonroutine clinical circumstances rather than a planned overnight observation pathway. December index admissions were excluded across all study years because the NRD cannot track readmissions across calendar years.

### Outcomes

The primary outcomes were all-cause readmission within 7 days and 30 days after discharge from the index LAAC hospitalization. For patients with multiple readmissions during the follow-up window, only the first readmission was analyzed. Secondary outcomes included cause-specific 30-day readmission and selected index-hospitalization complications.

Cause-specific readmission was classified using the principal diagnosis code of the first 30-day readmission. Categories were prespecified using clinically grouped ICD-10 codes informed by prior LAAC readmission literature.^15^ Code lists used to define comorbidities, index complications, and cause-specific readmission categories are provided in Supplementary Table 1.

### Covariates

Covariates were selected a priori based on clinical relevance and prior LAAC readmission studies.^10^, ^11^, ^12^^,^^15^ Patient-level covariates included age, sex, insurance, median household income quartile by patient zip code, and selected comorbidities. Comorbidities were derived from ICD-10 diagnosis codes and included hypertension, diabetes mellitus, heart failure, pulmonary circulation disease, peripheral vascular disease, chronic pulmonary disease, renal failure, liver disease, coagulopathy, obesity, anemia, and depression.

Hospital-level covariates included hospital bed size, hospital urban/teaching status, and hospital LAAC volume quartile. Hospital LAAC volume was calculated within each calendar year and categorized into year-specific quartiles. Calendar year was included to account for temporal changes in LAAC practice and discharge pathways.

### Statistical Analysis

Baseline characteristics were summarized according to discharge strategy. Categorical variables were reported as counts and percentages, and continuous variables were reported as mean with SD or median with interquartile range, as appropriate. Standardized mean differences were used to compare baseline characteristics. Missingness was low for model covariates; the primary multivariable analysis used complete-case analysis. A sensitivity analysis retained missing categorical covariates using a separate missing category. Temporal trends in SDD use were evaluated across calendar years.

Survey-weighted modified Poisson regression was used to estimate adjusted risk ratios (RRs) and 95% CIs for readmission associated with SDD versus overnight observation.^16^ Models incorporated NRD discharge weights and NRD strata, with year-specific hospital-clustered SEs to account for within-hospital correlation, and year-specific hospital identifiers were used because NRD hospital identifiers are not linkable across years. Calendar year was modeled using categorical indicators in outcome models. Temporal trends in SDD adoption were tested using survey-weighted Poisson regression with calendar year as a continuous variable.

Two models were examined. The first model adjusted for age, sex, and calendar year. The fully adjusted model additionally included insurance, income quartile, hospital characteristics, hospital LAAC volume quartile, and selected comorbidities. To quantify the potential influence of unmeasured confounding, e-values were calculated for the upper confidence limits of the primary 7-day and 30-day readmission estimates.^17^ We evaluated whether hospital LAAC volume modified the association between SDD and readmission using an SDD-by-volume quartile interaction term. Sensitivity analyses included a missing category approach for categorical covariates, propensity score overlap weighting,^18^ and an analysis adding LOS of at least 2 days as a third discharge duration category. Cause-specific readmission analyses were considered exploratory because of smaller event counts within individual categories. Exploratory cause-specific models used Firth penalized logistic regression with a reduced prespecified covariate set selected based on clinical relevance and parsimony given sparse event counts.^19^ The reduced model included age, sex, calendar year, insurance, hospital LAAC volume quartile, heart failure, renal failure, anemia, and coagulopathy.

## Results

### Study Cohort and Baseline Characteristics

A total of 40,267 LAAC hospitalizations with LOS 0 or 1 day were identified, including 1643 SDD hospitalizations and 38,624 overnight observation hospitalizations. Missingness was low: insurance was missing in 54 patients (0.13%), and zip income quartile was missing in 510 patients (1.27%) (Supplementary Table 2). The complete case analytic cohort included 39,705 hospitalizations, including 1621 SDD hospitalizations and 38,084 overnight observation hospitalizations. Overall, SDD accounted for 4.1% of the cohort. Baseline age was similar between groups: 76.1 ± 8.0 years in the SDD group versus 76.2 ± 7.8 years in the overnight stay group. Women represented 36.8% of the SDD group and 40.1% of the overnight stay group. Comorbidity burden, including heart failure, diabetes mellitus, renal failure, chronic pulmonary disease, and hypertension, was generally similar between groups. SDD was more frequent among patients treated at higher-volume LAAC hospitals and metropolitan teaching hospitals (Table 1).

**Table 1:** Baseline characteristics of patients undergoing same-day discharge versus overnight stay after left atrial appendage closure

Variable	SDD (n = 1621)	Overnight stay (n = 38,084)	SMD
Age, years	76.1 ± 8.0	76.2 ± 7.8	0.02
Female sex	597 (36.8)	15,279 (40.1)	0.07
Comorbidities			
Hypertension	1382 (85.3)	32,974 (86.6)	0.04
Diabetes mellitus	514 (31.7)	13,140 (34.5)	0.06
Heart failure	519 (32.0)	12,679 (33.3)	0.03
Pulmonary circulation disease	79 (4.9)	2283 (6.0)	0.05
Peripheral vascular disease	265 (16.3)	6450 (16.9)	0.02
Chronic pulmonary disease	323 (19.9)	8044 (21.1)	0.03
Renal failure	359 (22.1)	8576 (22.5)	0.01
Liver disease	42 (2.6)	964 (2.5)	0.00
Coagulopathy	41 (2.5)	1288 (3.4)	0.05
Obesity	268 (16.5)	6487 (17.0)	0.01
Anemia	214 (13.2)	6083 (16.0)	0.08
Depression	108 (6.7)	2797 (7.3)	0.03
Insurance			
Medicare	1483 (91.5)	34,227 (89.9)	0.06
Medicaid	15 (0.9)	390 (1.0)	0.01
Private insurance	104 (6.4)	2807 (7.4)	0.04
Other/self-pay	19 (1.2)	660 (1.7)	0.05
ZIP income quartile			
Quartile 1, lowest	309 (19.1)	7918 (20.8)	0.04
Quartile 2	393 (24.2)	10,062 (26.4)	0.05
Quartile 3	410 (25.3)	10,748 (28.2)	0.07
Quartile 4, highest	509 (31.4)	9356 (24.6)	0.15
Hospital LAAC volume quartile			
Quartile 1, lowest	51 (3.1)	1862 (4.9)	0.09
Quartile 2	132 (8.1)	5179 (13.6)	0.18
Quartile 3	261 (16.1)	9247 (24.3)	0.20
Quartile 4, highest	1177 (72.6)	21,796 (57.2)	0.33
Hospital bed size			
Small	31 (1.9)	2404 (6.3)	0.22
Medium	591 (36.5)	8839 (23.2)	0.29
Large	999 (61.6)	26,841 (70.5)	0.19
Hospital location/teaching status			
Metropolitan nonteaching	84 (5.2)	4863 (12.8)	0.27
Metropolitan teaching	1496 (92.3)	32,741 (86.0)	0.20
Nonmetropolitan	41 (2.5)	480 (1.3)	0.09

Values are mean ± SD or n (%).

Abbreviations: LAAC, left atrial appendage closure; SDD, same-day discharge; SMD, standardized mean difference.

### Temporal Trends in Same-Day Discharge

**Figure 1:**
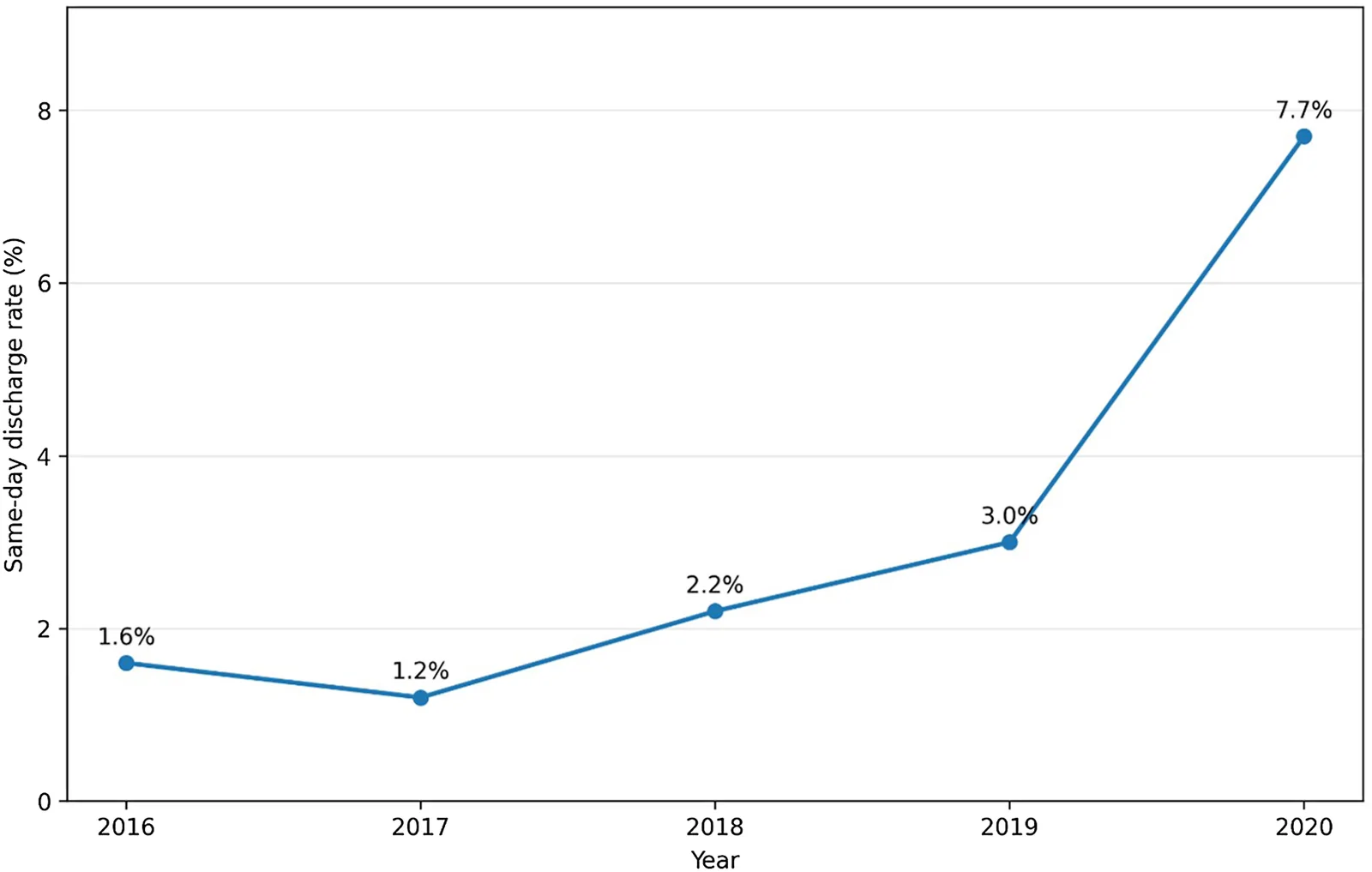
Temporal trends in same-day discharge after left atrial appendage closure, 2016–2020.

The use of SDD increased during the study period. SDD accounted for 1.6% of eligible LAAC hospitalizations in 2016, 1.2% in 2017, 2.2% in 2018, 3.0% in 2019, and 7.7% in 2020 (Figure 1). In formal trend testing, SDD use increased significantly over time (RR per year: 1.85; 95% CI 1.51–2.25; *p* < 0.001).

### All-Cause Readmission

Crude 7-day readmission occurred in 33 patients in the SDD group and 918 patients in the overnight stay group, corresponding to rates of 2.0 and 2.4%, respectively. In the fully adjusted survey-weighted model, SDD was not associated with higher 7-day readmission compared with overnight stay (RR 0.89; 95% CI 0.62–1.29; *p* = 0.549).

**Figure 2:**
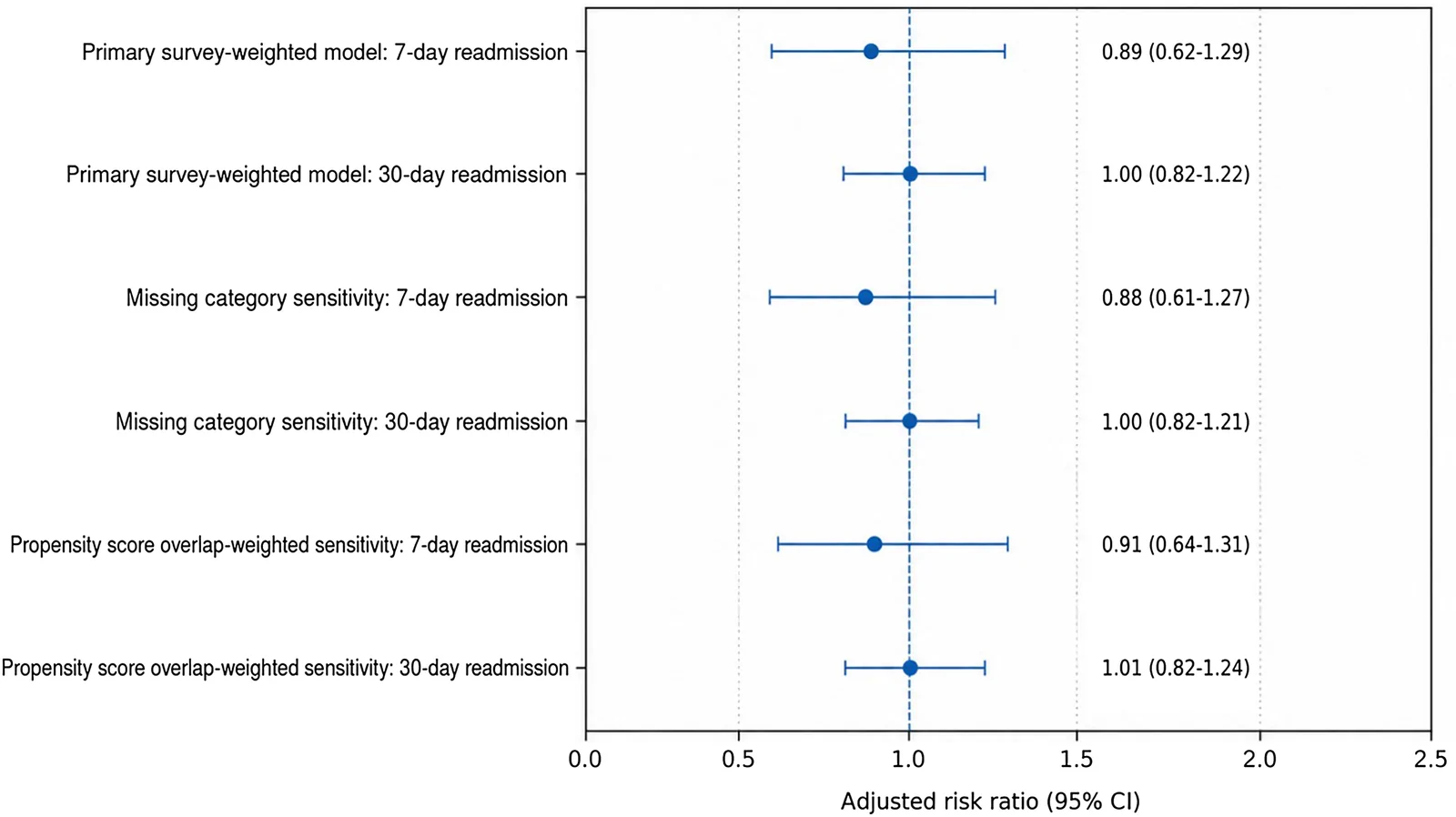
**Adjusted risk ratios for 7-day and 30-day readmission after same-day discharge versus overnight stay.** Primary estimates were derived from survey-weighted modified Poisson regression. Sensitivity analyses included missing category handling for categorical covariates and propensity score overlap weighting.

Crude 30-day readmission occurred in 115 patients in the SDD group and 2907 patients in the overnight stay group, corresponding to rates of 7.1 and 7.6%, respectively. SDD was not associated with higher 30-day readmission in the fully adjusted survey-weighted model (RR 1.00; 95% CI 0.82–1.22; *p* = 0.994) (Figure 2). Results were similar in the missing category sensitivity analysis and in the propensity score overlap–weighted sensitivity analysis (Table 2). Covariate balance improved after overlap weighting, with all postweighting standardized mean differences below 0.10 (Supplementary Table 3). The e-values corresponding to the upper confidence limits were 1.90 for 7-day readmission and 1.74 for 30-day readmission, indicating that unmeasured confounding of modest magnitude could account for the upper bound estimates compatible with increased readmission risk.

**Table 2:** Association between same-day discharge and early readmission after left atrial appendage closure

Analysis	Outcomes	SDD (n = 1621)	Overnight stay (n = 38,084)	Adjusted RR (95% CI)	*p* Value
Primary survey-weighted model	7-d readmission	33 (2.0)	918 (2.4)	0.89 (0.62-1.29)	0.549
	30-d readmission	115 (7.1)	2907 (7.6)	1.00 (0.82-1.22)	0.994
Missing category sensitivity	7-d readmission	33 (2.0)	934 (2.4)	0.88 (0.61-1.27)	0.488
	30-d readmission	116 (7.1)	2948 (7.6)	1.00 (0.82-1.21)	0.964
Propensity score overlap-weighted sensitivity	7-d readmission	-	-	0.91 (0.64-1.31)	0.625
	30-d readmission	-	-	1.01 (0.82-1.24)	0.922

Primary model was a survey-weighted modified Poisson model using complete-case analysis. The fully adjusted model included age, sex, calendar year, hypertension, diabetes mellitus, heart failure, pulmonary circulation disease, peripheral vascular disease, chronic pulmonary disease, renal failure, liver disease, coagulopathy, obesity, anemia, depression, insurance, income quartile, and hospital characteristics. Missing category sensitivity analysis retained missing values in categorical covariates as a separate category.

Abbreviations: RR, risk ratio; SDD, same-day discharge.

### Index Hospitalization Complications

Selected index hospitalization complications are shown in Table 3. Bleeding and pericardial complications were numerically less frequent among SDD patients than among patients observed overnight. In-hospital death, stroke or transient ischemic attack, and acute kidney injury were rare in the SDD group.

**Table 3:** Index hospitalization complications by discharge strategy

Index hospitalization event	SDD (n = 1621)	Overnight stay (n = 38,084)
Bleeding/transfusion	60 (3.7)	1995 (5.2)
Pericardial effusion/tamponade/pericardiocentesis	16 (1.0)	645 (1.7)
Stroke/transient ischemic attack	≤10	173 (0.5)
Vascular/access complication	14 (0.9)	323 (0.8)
Acute kidney injury	≤10	251 (0.7)
In-hospital death	≤10	≤10

Events were identified using ICD-10 codes during the index hospitalization. Cells with 10 or fewer events were suppressed in accordance with HCUP reporting standards.

Abbreviations: HCUP, Healthcare Cost and Utilization Project; SDD, same-day discharge; ICD, International Classification of Diseases.

### Hospital LAAC Volume

SDD was more common at higher-volume LAAC hospitals. SDD occurred in 51 of 1913 hospitalizations (2.7%) in the lowest hospital-volume quartile and 1177 of 22,973 hospitalizations (5.1%) in the highest quartile (Table 4). There was no statistically significant interaction between SDD and hospital LAAC volume quartile for 7-day readmission (*p* for interaction = 0.773) or 30-day readmission (*p* for interaction = 0.454).

**Table 4:** Readmission outcomes by discharge strategy and hospital LAAC volume quartile

Hospital LAAC volume quartile	Total cases	SDD, n (%)	Overnight stay, n (%)	7-d readmission after SDD	7-d readmission after overnight stay	30-d readmission after SDD	30-d readmission after overnight stay
Quartile 1, lowest	1913	51 (2.7)	1862 (97.3)	≤10	57 (3.1)	≤10	180 (9.7)
Quartile 2	5311	132 (2.5)	5179 (97.5)	≤10	150 (2.9)	≤10	434 (8.4)
Quartile 3	9508	261 (2.7)	9247 (97.3)	≤10	238 (2.6)	16 (6.1)	726 (7.9)
Quartile 4, highest	22,973	1177 (5.1)	21,796 (94.9)	25 (2.1)	473 (2.2)	86 (7.3)	1567 (7.2)

Hospital LAAC volume was calculated within each calendar year and categorized into year-specific quartiles. Cells with 10 or fewer events were suppressed in accordance with HCUP reporting standards. In adjusted interaction testing, there was no statistically significant interaction between SDD and hospital LAAC volume quartile for 7-d readmission (*p* for interaction = 0.773) or 30-d readmission (*p* for interaction = 0.454).

Abbreviations: HCUP, Healthcare Cost and Utilization Project; LAAC, left atrial appendage closure; SDD, same-day discharge.

### Cause-Specific Readmission and LOS Sensitivity Analysis

Among 3022 30-day readmissions, the most common principal diagnosis categories were bleeding/anemia, cardiovascular/thromboembolic causes including heart failure/volume overload, infection/sepsis, and arrhythmia/conduction disorders (Supplementary Table 4). Cause-specific readmission distributions were broadly similar between SDD and overnight-observation groups. Exploratory Firth penalized logistic regression models using a reduced covariate set showed no signal of excess cause-specific readmission after SDD (Supplementary Table 5).

In a sensitivity analysis adding LOS of at least 2 days as a third category, patients with LOS of at least 2 days had substantially higher readmission rates than the overnight stay group. Compared with LOS 1, LOS of at least 2 days was associated with higher 7-day readmission (RR 1.90; 95% CI 1.66–2.18) and 30-day readmission (RR 1.72; 95% CI 1.59–1.86), whereas the LOS 0 versus LOS 1 comparison remained similar to the primary analysis (Supplementary Table 6).

## Discussion

In this national analysis of LAAC hospitalizations from 2016 through 2020, several clinically relevant findings emerged. First, SDD increased substantially over time but remained uncommon overall. Second, among patients discharged after LOS 0 or LOS 1, SDD was not associated with higher 7-day or 30-day readmission compared with overnight observation in survey-weighted multivariable models. Third, this finding was consistent in missing category and propensity score overlap–weighted sensitivity analyses. Fourth, SDD was more common at higher-volume LAAC hospitals, but the association between SDD and readmission did not differ significantly by hospital LAAC volume quartile. Finally, cause-specific readmission patterns were broadly similar between SDD and overnight stay groups. Importantly, these findings should be interpreted as outcomes among patients selected for SDD rather than evidence that SDD is universally safe for all patients undergoing LAAC.

Our findings should be interpreted within the broader evolution of LAAC practice. Early randomized trials and long-term follow-up studies established the efficacy of LAAC for stroke prevention in selected patients with nonvalvular AF, whereas contemporary registry data have shown improved procedural safety in real-world practice.^4^, ^5^, ^6^, ^7^ This safety evolution provides the clinical rationale for reassessing traditional overnight observation after uncomplicated LAAC, particularly as structural heart programs increasingly emphasize streamlined recovery pathways, efficient resource use, and patient-centered postprocedural care.^2^^,^^7^

Prior NRD studies have supported the safety of SDD after LAAC, but important differences in comparator definitions remain. Maqsood et al.^10^ compared SDD with all later discharges, and Kawamura et al.^11^ compared SDD with any hospitalization lasting at least 1 night—approaches that may include patients with prolonged stays due to complications, delayed recovery, or greater clinical acuity.^10^^,^^11^ In contrast, Zahid et al.^12^ compared SDD with next-day discharge and found similar 30-day readmission rates with lower hospitalization costs. Our study builds on this literature by focusing on the clinically relevant decision between SDD and routine overnight observation while adding 7-day readmission as an early outcome, survey-weighted analyses, hospital-volume subgroup analyses, index hospitalization complications, and LOS ≥2 sensitivity analyses.

The absence of excess early readmission with SDD should be interpreted in the context of patient selection. Patients selected for SDD likely had a more favorable procedural course, fewer immediate postprocedural concerns, and sufficient home support. These factors are central to real-world SDD decision-making but are not fully captured in the NRD. Therefore, our findings should not be interpreted as evidence that all patients undergoing LAAC are candidates for SDD. Rather, they suggest that among patients selected for discharge after an uncomplicated early postprocedural course, SDD was not associated with a statistically significant increase in early readmission compared with overnight observation.

The increase in SDD during the study period may reflect several parallel changes in LAAC practice, including increasing operator experience, improvements in procedural workflow, and greater familiarity with postprocedural management. The marked increase in 2020 may also reflect pandemic-era pressure to reduce inpatient exposure and preserve hospital capacity, although our study was not designed to determine the causal drivers of this temporal shift. Because only 5 annual time points were available, we did not perform a formal interrupted time series analysis to isolate a pandemic effect; therefore, the 2020 increase should be interpreted as an association rather than evidence of a causal pandemic-related shift. Regardless of the underlying mechanism, the increasing adoption of SDD highlights the need for structured discharge criteria and postdischarge follow-up protocols within LAAC programs.

The cause-specific readmission findings provide practical insight for clinical pathway design. Prior LAAC readmission studies have identified bleeding, heart failure, infection, and arrhythmia as common reasons for readmission after LAAC.^11^^,^^15^ In our analysis, bleeding/anemia was the most common classifiable category among 30-day readmissions, followed by cardiovascular/thromboembolic causes including heart failure/volume overload, infection/sepsis, and arrhythmia/conduction disorders. Exploratory cause-specific models using Firth penalized logistic regression showed no signal of excess cause-specific readmission after SDD, although these analyses were limited by sparse events and should be interpreted cautiously. These findings suggest that reducing readmissions after LAAC may require attention not only to discharge timing, but also to postdischarge medication management, bleeding precautions, volume status, vascular access instructions, and early access to clinical support.

Hospital experience may influence adoption of SDD pathways. In our analysis, SDD was more common at higher-volume LAAC hospitals, suggesting that institutional experience, workflow, and comfort with postprocedural monitoring may influence discharge practice. However, we did not observe a statistically significant interaction between SDD and hospital LAAC volume quartile for 7-day or 30-day readmission. These findings suggest that hospital volume may influence the likelihood of SDD adoption, but we did not find evidence that the association between SDD and early readmission differed significantly across hospital-volume strata.

The LOS ≥2 sensitivity analysis further supports the use of LOS 1 as the primary comparator. Patients hospitalized for at least 2 days had substantially higher 7-day and 30-day readmission rates than patients discharged after overnight observation, consistent with a clinically distinct group with delayed recovery, complications, or greater illness severity. Prior NRD studies often compared SDD with broader non-SDD groups that included patients hospitalized beyond 1 day, whereas Zahid et al.^12^ specifically examined next-day discharge as a comparator.^10^^,^^11^ Therefore, combining LOS 1 and LOS ≥2 together may obscure the practical clinical question of SDD versus routine overnight observation.

This study has several limitations. First, the observational design precludes causal inference, and residual confounding by patient selection remains likely despite multivariable adjustment, survey weighting, and propensity score overlap–weighted sensitivity analysis. Patients selected for SDD may have had greater procedural stability, fewer intraprocedural concerns, or stronger social support than patients kept overnight, none of which are fully captured in the NRD. Consistent with the modest e-values, residual confounding from unmeasured factors such as procedural stability, frailty, recovery from anesthesia, and social support remains possible. Second, the primary cohort was restricted to patients with LOS 0 or 1 day and therefore reflects patients with an uncomplicated or early stabilized postprocedural course; the findings should not be generalized to all LAAC patients. Third, postdischarge death is not captured in the NRD and may represent an unmeasured competing risk for readmission. Fourth, this analysis is subject to limitations inherent to administrative NRD data. Diagnoses, procedures, comorbidities, index complications, and readmission causes were identified using administrative codes and may be subject to misclassification. Furthermore, because the NRD permits patient linkage within a calendar year but not across years, December index admissions were excluded for 30-day readmission assessment, and cross-year repeated procedures or within-patient correlation across years could not be assessed. Finally, although the analysis included data through 2020, more contemporary practice patterns after broader adoption of newer LAAC devices and evolving discharge protocols may differ.

In conclusion, in a national cohort of patients undergoing LAAC from 2016 through 2020, SDD increased over time but remained uncommon. Among patients selected for discharge after LOS 0 or LOS 1, SDD was not associated with a statistically significant increase in 7-day or 30-day readmission compared with overnight observation. These findings support the feasibility of carefully selected SDD pathways after uncomplicated LAAC, while emphasizing that SDD decisions should incorporate patient selection, procedural stability, and adequate postdischarge support. Prospective studies are needed to define optimal patient selection and postprocedural care pathways.

## Data Availability Statement

No new data were generated. The NRD data are available from HCUP/AHRQ to authorized users under a data use agreement.

## Ethics Statement

The research reported in this article adhered to the relevant ethical guidelines and was conducted in accordance with the principles outlined in the Declaration of Helsinki.

## Funding

The authors have no funding to report.

## Declaration of Generative AI and AI-Assisted Technologies in the Writing Process

The authors used ChatGPT for language editing and grammar refinement. The authors reviewed and edited all content and take full responsibility for the accuracy, integrity, interpretation, and originality of the manuscript.

## Disclosure Statement

The authors report no conflict of interest.
