# Supplementary material for: Same-Day Discharge After Left Atrial Appendage Closure and Early Readmissions: A 5-Year National Readmissions Database Analysis
Source: Struct Heart. 2026 Jun 24;10(9):101073. doi: 10.1016/j.shj.2026.101073 (PMC13616499; doi:10.1016/j.shj.2026.101073)
Supplement: Supplementary file 1 [file mmc1.docx]

Supplementary Table 1. ICD-10 Code Definitions

| Domain | Definition | ICD-10 code |
| --- | --- | --- |
| LAAC procedure | Percutaneous left atrial appendage closure | 02L73DK |
| Hypertension | Hypertension diagnosis | I10–I16 |
| Diabetes mellitus | Diabetes diagnosis | E08–E13 |
| Heart failure | Heart failure diagnosis | I50, I110, I130, I132 |
| Pulmonary circulation disease | Pulmonary embolism or pulmonary hypertension | I26, I27 |
| Peripheral vascular disease | Peripheral arterial/vascular disease | I70–I73, I771, I790, I792, K551, K558, K559, Z958, Z959 |
| Chronic pulmonary disease | Chronic obstructive or chronic lung disease | J40–J47, J60–J67, J684, J701, J703 |
| Renal failure | Acute or chronic kidney failure | N17–N19, I120, I131, I132, N250, Z49, Z940, Z992 |
| Liver disease | Chronic liver disease/cirrhosis-related diagnoses | B18, I85, K70–K77, Z944 |
| Coagulopathy | Coagulation disorder or thrombocytopenia | D65–D68, D691, D693–D696 |
| Obesity | Obesity diagnosis | E66 |
| Anemia | Anemia diagnosis | D50–D64 |
| Depression | Depression diagnosis | F32, F33, F341 |
| Bleeding/transfusion complication | Bleeding, hemorrhage, anemia, or transfusion during index hospitalization | D62, D50–D64, D683, K920–K922, R04, R31 |
| Pericardial complication | Pericardial effusion, hemopericardium, tamponade, or pericardiocentesis | I30, I31, I974 |
| Stroke/TIA | Ischemic stroke or transient ischemic attack | I63, I64, G45 |
| Vascular/access complication | Vascular injury, access-site complication, pseudoaneurysm, or procedural vascular complication | T81, T82, I97, I72, L76 |
| Acute kidney injury | Acute kidney injury during index hospitalization | N17 |
| **Cause-specific readmission** |  |  |
| Bleeding/anemia | Principal diagnosis of bleeding or anemia | D50–D64, D683, K920–K922, K25–K28 with hemorrhage, K31811, K5521, K57 with hemorrhage, K625, R04, R31, L7632 |
| Cardiovascular/thromboembolic including heart failure/volume overload | Principal diagnosis of heart failure, volume overload, ischemic/thromboembolic event, or other cardiovascular/symptom-based cause | I50, I110, I130, I132, E877, I21–I24, I26, I63, I64, G45, I82, R07, R55 |
| Infection/sepsis | Principal diagnosis of infection or sepsis | A40–A41, R65, J13–J18, N390, L03, U071 |
| Arrhythmia/conduction disorder | Principal diagnosis of arrhythmia or conduction disorder | I44–I49, R00 |
| Other classifiable causes | Gastrointestinal nonbleeding, pericardial/procedural/access, renal/metabolic, or respiratory noninfectious causes | K codes excluding bleeding categories, I30–I31, T81–T82, I97, N17–N19, E86–E87 excluding E877, J44–J96 excluding infectious pneumonia |
| Other/uncategorized | Principal diagnosis not otherwise classified | Remaining ICD-10 principal diagnosis codes |

Supplementary Table 2. Missingness of Model Covariates

| Variable/domain | Missing, n(%) |
| --- | --- |
| Age | 0 (0.0) |
| Sex | 0 (0.0) |
| ICD-10 code derived comorbidities | 0 (0.0) |
| Insurance | 54 (0.13) |
| ZIP income quartile | 510 (1.27) |
| Hospital LAAC volume quartile | 0 (0.0) |
| Hospital bed size | 0 (0.0) |
| Hospital urban/teaching status | 0 (0.0) |

Study cohort included 40,267 hospitalizations. The primary multivariable analysis used complete-case analysis. A sensitivity analysis retained missing categorical covariates using a separate missing category. LAAC indicates left atrial appendage closure

Supplementary Table 3. Covariate Balance After Propensity Score Overlap Weighting

| Variable | Overlap-weighted  SDD | Overlap-weighted  Overnight stay | SMD |
| --- | --- | --- | --- |
| Age, years | 76.1 ± 8.0 | 76.1 ± 7.8 | 0.001 |
| Female sex | 37.0 | 37.1 | 0.001 |
| **Comorbidities, %** |  |  |  |
| Hypertension | 85.3 | 85.4 | 0.001 |
| Diabetes mellitus | 32.0 | 32.0 | 0.000 |
| Heart failure | 32.3 | 32.3 | 0.000 |
| Pulmonary circulation disease | 5.0 | 5.0 | 0.000 |
| Peripheral vascular disease | 14.1 | 14.1 | 0.000 |
| Chronic pulmonary disease | 19.7 | 19.7 | 0.000 |
| Renal failure | 22.6 | 22.6 | 0.000 |
| Liver disease | 3.0 | 2.9 | 0.002 |
| Coagulopathy | 2.6 | 2.7 | 0.006 |
| Obesity | 16.6 | 16.6 | 0.000 |
| Anemia | 13.4 | 13.4 | 0.001 |
| Depression | 6.9 | 6.9 | 0.001 |
| **Insurance** |  |  |  |
| Medicare | 91.4 | 91.2 | 0.006 |
| Medicaid | 0.9 | 0.9 | 0.001 |
| Private insurance | 6.4 | 6.5 | 0.002 |
| Other/self-pay | 1.1 | 1.2 | 0.010 |
| **ZIP income quartile** |  |  |  |
| Quartile 1, lowest | 19.1 | 19.1 | 0.005 |
| Quartile 2 | 24.5 | 24.5 | 0.002 |
| Quartile 3 | 25.7 | 25.7 | 0.002 |
| Quartile 4, highest | 30.7 | 30.7 | 0.001 |
| **Hospital LAAC volume quartile** |  |  |  |
| Quartile 1, lowest | 3.3 | 3.2 | 0.007 |
| Quartile 2 | 8.5 | 8.5 | 0.001 |
| Quartile 3 | 16.8 | 16.8 | 0.002 |
| Quartile 4, highest | 71.4 | 71.5 | 0.001 |
| **Hospital bed size** |  |  |  |
| Small | 2.0 | 2.1 | 0.007 |
| Medium | 34.9 | 34.8 | 0.002 |
| Large | 63.1 | 63.0 | 0.002 |
| **Hospital location/teaching status** |  |  |  |
| Metropolitan nonteaching | 5.5 | 5.7 | 0.008 |
| Metropolitan teaching | 92.2 | 92.2 | 0.002 |
| Nonmetropolitan | 2.3 | 2.2 | 0.010 |
| **Calendar year, %** |  |  |  |
| 2016 | 2.4 | 2.7 | 0.016 |
| 2017 | 3.6 | 3.5 | 0.003 |
| 2018 | 10.8 | 10.8 | 0.001 |
| 2019 | 23.7 | 23.7 | 0.001 |
| 2020 | 59.5 | 59.3 | 0.003 |

Values are weighted means for age and weighted percentages for categorical variables in the overlap-weighted population. Propensity scores for same-day discharge were estimated using measured demographic, clinical, socioeconomic, hospital-level, and calendar-year covariates. LAAC indicates left atrial appendage closure; SDD, same-day discharge; SMD, standardized mean difference.

Supplementary Table 4. Cause-specific 30-day readmission distribution after left atrial appendage closure

| Cause-specific readmission category | 30-day readmissions, n(%) |
| --- | --- |
| Bleeding/anemia | 678 (22.4) |
| Cardiovascular/thromboembolic including heart failure/volume overload | 520 (17.2) |
| Infection/sepsis | 291 (9.6) |
| Arrhythmia/conduction disorder | 264 (8.7) |
| Other classifiable causes | 569 (18.8) |
| Other/uncategorized | 700 (23.2) |

Supplementary Table 5. Exploratory Firth Penalized Logistic Regression Models for Cause-Specific 30-Day Readmission

| Cause-specific 30-day readmission | SDD  (n=1,621) | Overnight stay  (n=38,084) | Firth OR  (95% CI) | P value |
| --- | --- | --- | --- | --- |
| Bleeding/anemia | 29 (1.8) | 649 (1.7) | 1.15 (0.79-1.68) | 0.458 |
| Cardiovascular/thromboembolic including heart failure/volume overload | 21 (1.3) | 499 (1.3) | 1.07 (0.69-1.66) | 0.762 |
| Infection/sepsis | 14 (0.9) | 277 (0.7) | 1.28 (0.75-2.18) | 0.360 |
| Arrhythmia/conduction disorder | 11 (0.7) | 253 (0.7) | 1.16 (0.64-2.10) | 0.629 |

Cause-specific outcomes were defined as first 30-day readmission with the listed principal diagnosis category among all index LAAC hospitalizations. Cardiovascular/thromboembolic causes include heart failure/volume overload, ischemic/thromboembolic events, and other cardiovascular/symptom-based causes. Firth penalized logistic regression was used because of sparse event counts in cause-specific categories, models adjusted for age, sex, calendar year, insurance, hospital LAAC volume quartile, heart failure, renal failure, anemia, and coagulopathy. CI indicates confidence interval; OR, odds ratio; SDD, same-day discharge.

Supplementary Table 6. Sensitivity Analysis Including LOS ≥2 Days as a Third Discharge Duration Category

| LOS | N | 7-day readmission, n (%) | 30-day readmission,  n (%) | Adjusted RR for  7-day readmission  (95% CI) | Adjusted RR for  30-day readmission  (95% CI) |
| --- | --- | --- | --- | --- | --- |
| LOS 0 | 1,621 | 33 (2.0) | 115 (7.1) | 0.87 (0.60-1.25) | 0.99 (0.81-1.21) |
| LOS 1 | 38,084 | 918 (2.4) | 2,907 (7.6) | Reference | Reference |
| LOS ≥2 | 5,412 | 334 (6.2) | 933 (17.2) | 1.90 (1.66-2.18) | 1.72 (1.59-1.86) |

Models adjusted for age, sex, calendar year, hypertension, diabetes mellitus, heart failure, pulmonary circulation disease, peripheral vascular disease, chronic pulmonary disease, renal failure, liver disease, coagulopathy, obesity, anemia, depression, insurance, income quartile, and hospital characteristics. LOS indicates length of stay; RR, risk ratio; CI, confidence interval.
